# Prevalence, genetic diversity, and molecular detection of the apple hammerhead viroid in Germany

**DOI:** 10.3389/fmicb.2025.1592572

**Published:** 2025-06-03

**Authors:** Kerstin Zikeli, Constanze Berwarth, Ute Born, Thomas Leible, Wilhelm Jelkmann, Michael Helmut Hagemann

**Affiliations:** ^1^Julius Kühn-Institute, Federal Research Centre for Cultivated Plants, Institute for Plant Protection in Fruit Crops and Viticulture, Dossenheim, Germany; ^2^Department of Production Systems of Horticultural Crops, University of Hohenheim, Stuttgart, Germany

**Keywords:** *Avsunviroidae*, next-generation sequencing (NGS) technologies, RNA plant pathogens, molecular plant diagnostics, viroid variability

## Abstract

**Introduction:**

Apple hammerhead viroid (AHVd) is an emerging plant pathogen infecting apple orchards worldwide. Its genetic variability and geographical distribution remain poorly understood, limiting effective diagnostics and management strategies.

**Methods:**

In this study, 192 samples from German apple orchards were analyzed using reverse transcription (RT) and real-time PCR, one-step RT real-time PCR, and Sanger sequencing. Next-generation sequencing (NGS) was employed on pooled RNA extracts to explore genetic diversity. Phylogenetic relationships were inferred using maximum likelihood methods, and viroid-derived small RNAs (vd-sRNAs) were identified from small RNA sequencing data.

**Results and discussion:**

AHVd was detected in 78% of samples, with prevalence varying by region: southern (82%), eastern (90%), northern (72%), and western (70%) states of Germany. Phylogenetic analysis revealed distinct clusters linked to geographical origins, indicating isolated evolutionary pathways. NGS analysis uncovered 39% inter-sample variability and 169 polymorphic positions, while Sanger sequencing of RT real-time PCR products derived from the same samples showed only 3% variability, reflecting dominant quasispecies populations. Small RNA analysis mapped 128,388 reads to the AHVd genome, identifying hotspots within and outside the rod-like structure, suggesting structural and regulatory functions of vd-sRNAs. These findings underline AHVd’s genetic diversity. The complex relationship between AHVd genetic variability and symptom expression necessitates the development of highly sensitive diagnostic tools and adaptive management strategies to effectively monitor and control its spread in apple production.

## Introduction

Viroids are small, circular, single-stranded RNA molecules that infect plants, causing various diseases. Unlike viruses, viroids do not encode proteins but replicate autonomously in host plants, often leading to significant agricultural losses. The viroid family is divided into two major groups: *Pospiviroidae* and *Avsunviroidae*. Members of the *Pospiviroidae* family have a rod-like structure and replicate in the nucleus, while *Avsunviroidae* viroids have a branched structure and replicate in plastids, featuring self-cleaving hammerhead ribozymes involved in their replication mechanism (Di Serio et al., 2018). The *Avsunviroidae* family includes genera such as *Avsunviroid*, including only the avocado sunblotch viroid (ASBVd, *Avsunviroid albamaculaperseae*), the *Elaviroid* including only the eggplant latent viroid (ELVd, *Elaviroid latensmelongenae*), and the *Pelamoviroid*, with the members chrysanthemum chlorotic mottle viroid (CChMVd, *Pelamoviroid maculachrysanthemi*), peach latent mosaic viroid (PLMVd, *Pelamoviroid latenspruni*), and the latest addition, the apple hammerhead viroid (AHVd, *Pelamoviroid malleusmali*, Di Serio et al., 2018).

For ELVd it has already been shown that the mutation rate and consequently the inter-sample variability is much higher compared to viroids from the *Pospiviroidae*, as determined by high-fidelity ultra-deep sequencing (López-Carrasco et al., 2017). Furthermore, the inter-sample variability of PLMVd was observed to include 3,939 different sequences within a single infected peach tree (Glouzon et al., 2014). Despite this variability, approximately 50% of the positions in PLMVd remained conserved in that study. Similar studies on the AHVd are currently lacking. The first report to identify the apple hammerhead viroid-like RNA (AHVd-like RNA) was published in 2014 using advanced RNA sequencing and computational algorithms (Zhang et al., 2014). Subsequent studies associated this RNA with different symptoms and further observed high sequence variability. For instance, the sequence identity between Italian variants and previously reported variants from other regions (China, Canada, and USA) ranged from 82 to 88% (Chiumenti et al., 2019). This high genetic variability poses a challenge for diagnostic screening, as the efficiency and reliability of primer-probe combinations used in RT real-time PCR and Sanger sequencing methods can be affected. Nabi and Baranwal (2020) reported the presence of AHVd in symptomatic apple cultivars (*Malus domestica* L.) in India, showing mosaic, ringspot, and necrosis symptoms. However, the role of AHVd in symptom expression remains unclear, as co-infection with other viruses or viroids may influence symptoms, or certain AHVd variants may be symptom-inducing while others remain latent. Similarly, Szostek et al. (2018) found AHVd in both symptomatic (e.g., shoot decline and dieback) and asymptomatic apple trees across various countries, highlighting the diverse symptomatology in apple, ranging from fruit quality impairments to severe destructive symptoms, and emphasizing the difficulty in correlating specific symptoms with the presence of AHVd. AHVd has been reported in apple cultivars from diverse geographical regions, including China, Canada, the United States, Japan, Italy, Spain, Montenegro, South Korea, and India (Zhang et al., 2014; Messmer et al., 2017; Szostek et al., 2018; Lim et al., 2019; Nabi and Baranwal, 2020; Zindović et al., 2024). In 2020, AHVd was first reported in Germany (Zikeli et al., 2021). Despite its widespread distribution, studies have found no consistent correlation between the presence of AHVd and specific symptoms or poor growth (Lim et al., 2019). For instance, Serra et al. (2018) reported considerable sequence diversity among AHVd isolates, with the reference sequence of variant “Pacific Gala” differing by 62 positions (86% identity) from “Fuji.” This diversity complicates efforts to trace the origin and spread of AHVd infections.

The secondary structure of viroids plays a crucial role in their replication and pathogenicity. AHVd, like other members of the *Pelamoviroid* genus, adopts a branched conformation stabilized by a kissing-loop interaction (Chiumenti et al., 2019). Despite the high sequence variability, critical elements of the hammerhead ribozymes, which are essential for replication, remain conserved (Serra et al., 2018). The mutation rate of *Avsunviroidae* members, including AHVd, is among the highest of any biological entity, likely due to replication by a proofreading-deficient RNA polymerase (López-Carrasco et al., 2017; Flores et al., 2017). This high mutation rate leads to significant genetic diversity within the viroid population, as observed in PLMVd and ELVd (Glouzon et al., 2014). Understanding the impact of these variable regions on the secondary structure of AHVd is crucial for elucidating its pathogenic mechanisms and for developing effective diagnostic tools.

The aims of this study were to investigate the prevalence of AHVd in Germany, the high genetic variability of AHVd and its impact on diagnostic screening and geographical distribution. We hypothesized that the genetic variability of AHVd leads to inconsistent screening results due to observed differences in obtained results of primer-probe combinations used in different PCR and sequencing methods. Additionally, we aimed to explore whether specific sequence variants of AHVd correlate with distinct geographical regions, suggesting unique infection sources or transmission pathways.

## Materials and methods

### Plant material

In 2022, the Julius Kühn-Institute (JKI) initiated a nationwide survey, collecting over 1,000 apple shoot samples from 41 locations across 14 German federal states, representing more than 200 apple cultivars. Samples were obtained from commercial and scattered orchards, nurseries, trial farms, and variety collections between May and July 2022. The samples were originally intended to be processed independently in two laboratories (JKI and the University of Hohenheim, UHOH) using standardized RT real-time PCR detection protocols. However, after more than 500 PCR attempts using different cDNA preparations, primer and probe combinations, and amplification platforms, the results remained inconsistent and difficult to interpret. These challenges highlighted the limitations of existing detection methods, particularly in light of the viroid’s high sequence variability.

In response, new primers were designed using the Primer3 tool integrated in Geneious Prime^®^ (version 2022.1.1, Biomatters Ltd., New Zealand), based on an alignment of all 109 full-length AHVd sequences available in GenBank as of August 14, 2024. To ensure robustness, we adopted a two-step diagnostic approach: RT real-time PCR for initial template enrichment, followed by Sanger sequencing of all final amplicons, regardless of curve quality. Out of the over 1,000 apple samples a representative subset of 192 samples was selected for parallel testing in both laboratories. Total RNA was extracted from phloem scrapings as described below. Each sample was analyzed using two RT real-time PCR assays (Table 1), and the resulting products were sequenced to reliably determine AHVd presence and variation. To further investigate viroid diversity, total RNA sequencing was conducted at UHOH on pooled screening samples. In addition, small RNA sequencing was performed on a single tree (cv. “Nicoter”/”Kanzi,” 15 years old) from the UHOH research station in Stuttgart-Plieningen. Leaf material was immediately shock-frozen in liquid nitrogen and stored at −80°C until further processing.

**Table 1:** List of primers and probes used in this study.

Type	Primer/Probe	Sequence	Author
Real-time PCR ZK	AHVd-13F_PG	CCTTCCTGATGAGTCCGTTCCA	Messmer et al. (2017)
	AHVd-2	TGTGTCTACTTAAAGACTCAC	Lim et al. (2019)
	AHVd-ZK-P_HEX	(HEX)TCATCAGGTAGCCTAATAGACTAC(BHQ1)	This publication
Real-time PCR MHX	AHVd-MHx-F	ACCCCTCCGGTCTTATCCAA	This publication
	AHVd-MHx-R	CGTCCTTGGAACGGACTCAT	This publication
	AHVd-MHx-P-FAM	(FAM)GACGGTCGGAGGCTATTAGC(BHQ1)	This publication

### Total RNA extraction and viroid amplification

For different PCR methods total RNA was extracted from shoot samples using a CTAB extraction method (adapted from Carra et al., 2007). Briefly, 100 mg of scraped phloem was ground in liquid nitrogen and mixed with 950 μL CTAB extraction buffer (prewarmed at 60°, containing 0.2% β-mercaptoethanol) by vortexing for 2 min. The sample was incubated at 60°C for 5 min and extracted by adding an equal volume of chloroform and shaking for 2 min. After centrifugation at 10,000 rpm for 10 min at 4°C the aqueous phase was transferred to a fresh tube. This chloroform extraction step was performed twice. To the aqueous phase, 0.8 volume of cold isopropanol was added, mixed by gentle inversion and RNA was precipitated overnight at −20°C. The sample was centrifuged at 13,000 rpm for 30 min at 4°C and the pellet was washed with 70% ethanol. Finally, the dried pellet was resolved in 100 μL of RNase-free water and stored at −80°C.

Success of the extraction was confirmed by analysing RNA extracts via internal control RT-PCR using the primers Plum_Actin7_1137F and Plum_Actin7_1610R which target an actin-7 mRNA, which is conserved in members of the Rosaceae family (Bester et al., 2020). Two complementary RT real-time PCR assays were performed for each sample to amplify regions of AHVd genome. The primers were designed based on conserved regions of AHVd, allowing for up to three mismatches to account for sequence variability. Therefore, the mentioned NCBI full-length AHVd accessions (*n* = 109) were utilized. The first RT real-time PCR employed the primers AHVd-13F_PG (forward) and AHVd-2 (reverse) along with the probe AHVd-ZK_P-HEX (Table 1), generating an amplicon of 373 nt based on the reference sequence NC_028132. The second RT real-time PCR used the primers AHVd-MHx-F and AHVd-MHx-R along with the probe AHVd-MHx_P-FAM (Table 1), producing a complementary 250 nt amplicon, allowing both amplicons to be assembled into a nearly full-length AHVd genome leaving a 14-nt gap after trimming in all cases.

The RT real-time PCR was conducted with the Bioline SensiFast Probe No-Rox One Step kit (Bioline, London, United Kingdom). Each reaction consisted of 10 μL of mastermix (2×), 6.6 μL RNase-free water, 0.8 μL per primer (10 μM), 0.2 μL of probe (10 μM), 0.2 μL reverse transcriptase, 0.4 μL Ribosafe RNase Inhibitor, and 1 μL RNA template in a total volume of 20 μL. The thermal cycling conditions were as follows: reverse transcription at 45°C for 10 min, initial denaturation at 95°C for 2 min, followed by 40 cycles of 95°C for 5 s and 60°C for 20 s. Thermal cycling was performed on a Biorad CFX96 Touch Real-Time Cycler (Bio-Rad Laboratories GmbH, Feldkirchen, Germany) and a Rotorgene 6000 (Qiagen, Hilden, Germany). RT real-time PCR curves were not assessed, and all samples were sent for sequencing irrespective of the RT real-time PCR results. Pre-experiments indicated that reliable curve interpretation was not possible due to high amplicon diversity. Thus, RT real-time PCR served as an enrichment step prior to Sanger sequencing.

### Sanger sequencing

Amplicons of all RT real-time PCR samples were purified by Exo-CIP Rapid PCR Cleanup Kit (New England Biolabs, Ipswich, United States) with 3 μL each of Exo-CIP A and B, 5 μL of sample and 10 μL of deionised water. The mix was incubated at 37°C for 4 min and inactivated at 80°C for 1 min. Ten microlitres were submitted for sequencing. Sanger sequencing was performed for forward and reverse primer as a service at Genewiz (South Plainfield, New Jersey, United States). To bridge the 14-bp gap assembly further RT-PCRs were conducted with additional primers (Supplementary Table 1), which resulted in 52 true full-length sequences (NCBI accession numbers PQ683281-PQ683332). All resulting Sanger reads were imported as ab1-files into Geneious Prime, which was used for downstream analysis. Each amplicon was trimmed for its corresponding primer (allowing for up to three mismatches) and quality of 0.01 (Phred quality score of 20). Further, the reads were grouped per sample and mapped to the AHVd reference sequence accession NC_028132. For this purpose, the Geneious RNA mapper was used with the highest sensitivity settings and a consensus sequence was generated based on a threshold quality of 60%. The number of reads mapped to AHVd was counted and the presence of one or more successfully mapped reads was interpreted as successful identification of AHVd.

### Phylogenetical analysis

The 52 full-length accessions of AHVd from Germany were aligned along with the 109 curated full-length AHVd sequences mentioned earlier. Multiple sequence alignment was performed using MAFFT (v7.490) with the following settings: determination of the algorithm set to auto, scoring matrix set to 200PAM, gap opening penalty set to 3, offset value set to 1, and automatic determination of sequence direction (Katoh et al., 2002). The phylogenetic tree was constructed using the maximum likelihood method RAxML (v8.2.11) with the GTR GAMMA nucleotide model and the rapid hill-climbing algorithm. Branch support was assessed with 100 bootstrap replicates, starting from a completely random tree and using a parsimony-based random seed. The *Eriobotrya japonica* L. (loquat) sequence OK272503 was used as outgroup.

### Next generation sequencing of small and total RNA

Small RNA extraction was performed using the mirVana miRNA Isolation Kit (Thermo Scientific, London, United Kingdom). Initially, samples were weighed, and 10 volumes of Lysis/Binding Buffer were added per unit mass of tissue. The frozen tissue was ground to a powder with liquid nitrogen and mixed rapidly with the buffer. Subsequently, 1/10 volume of miRNA homogenate additive was added to the lysate, which was then vortexed, incubated on ice, and extracted with acid-phenol:chloroform. The upper aqueous phase was collected post-centrifugation. For small RNA enrichment, 1/3 volume of 100% ethanol was added to the aqueous phase, and the mixture was filtered using filter cartridges to bind large RNAs. The filtrate was collected, additional ethanol was added, and the mixture was filtered again. Wash solutions were applied, and the final RNA was eluted with pre-heated elution solution. The eluate containing the RNA was stored at −80°C. Next, the sample underwent ribo-depletion to remove abundant rRNA sequences prior to the cDNA library preparation and small RNA sequencing all conducted by a service provider (LGC Group, Berlin, Germany), using Illumina NextSeq 500/550 v2, generating 75 bp single-end reads. The small RNA sequencing data were submitted to the European Nucleotide Archive (ENA) under study accession PRJEB88384 (run accession ERR14838686).

Total RNA sequencing was performed on four pools, each containing 1 μL of RNA per sample from the 149 AHVd-positive samples out of the 192 initially screened via Sanger sequencing. These pools were grouped by federal region in Germany: 17 samples from northern states, 44 from southern, 35 from western, and 53 from eastern states. The four regional pools were sent to Macrogen Europe B.V. (Amsterdam, Netherlands) for library preparation and sequencing. The TruSeq Stranded Total RNA with Ribo-Zero Plant protocol was used, which includes a ribosomal RNA depletion step specifically optimized for plant RNA. Macrogen used the Illumina NovaSeq 6000 platform generating 150 bp paired-end reads. Downstream quality control steps included adapter trimming and removal of low-quality reads.

Adapter-clipped FASTQ files delivered from both NGS-providers were imported into Geneious Prime. Raw reads were first adapter-trimmed using the BBDuk tool within Geneious Prime. Subsequently, read normalization and error correction were performed using BBNorm (also within Geneious Prime), with the following settings: error correction enabled, target depth set to 100, and a minimum depth of 5. The small RNA data were filtered for small RNAs with a length between 18 and 30 nt for viroid-derived small RNA mapping. All data were mapped against the AHVd reference sequence (accession NC_028132). The mapping of reads was performed using different algorithms and settings: BBMap, Geneious, and Geneious RNA, each tested with medium, high, and highest sensitivity settings. The mapping results were compared to evaluate consistency and accuracy. Thereby, the Geneious RNA mapper set to high sensitivity provided a consistent consensus sequence with only a few ambiguous bases, indicating a higher confidence in the sequence alignment and representation. Variants were identified using the Geneious variant caller, with parameters set for a minimum coverage of 20, minimum variant frequency of 0.1, and individual polymorphisms annotated per position merged to one variant position, while ignoring the reference sequence to find only within-sample variation.

Primer-trimmed Sanger sequencing reads were initially processed with a 0.01 error rate and then combined into a single list. These reads were mapped to the NC_028132 reference of AHVd, and variant calling was performed using the same criteria as for the NGS data.

## Results

### Viroid screening

First, the consistency of RT real-time PCR results between labs was assessed, knowing from pre-experiments (data not shown) that this can be erratic. In this experiment, samples were considered AHVd-positive if the Cq value was ≤35. When comparing results from both RT real-time PCR assays conducted at “JKI” and “UHOH” labs, an agreement rate of 88.8% was observed. Similarly, the agreement rate between the RT real-time PCR assays “ZK” and “MHx” (Table 1), irrespective of the lab, was 86.7%.

Furthermore, the correlation between Sanger sequencing and RT real-time PCR results was assessed. If either lab found one or two Sanger reads to be AHVd positive, it was considered a positive result, and if both labs reported a positive result, it was deemed an agreement. Combining the results of both RT real-time PCR assays with Sanger sequencing, an overall agreement rate of 80.5% was achieved. Therefore, the results of the AHVd screening are based solely on the successfully identified AHVd Sanger reads. The results showed that out of the 192 samples, 149 (77.6%) were positive for AHVd, while 43 (22.4%) were negative (Table 2). The states have been grouped according to their location, whereas Bavaria and Baden-Württemberg are South, Hesse, Saarland, North Rhine-Westphalia, and Rhineland-Palatinate are West, Saxony-Anhalt, Mecklenburg-Western Pomerania, Thuringia, Brandenburg, and Saxony are East, and Lower Saxony, Schleswig-Holstein, and Hamburg are North. Comparing the southern states with the northern states, there is a slightly higher positivity rate in the South (81.5% vs. 72.0%). Further, the eastern states show a higher positivity rate (89.5%) compared to the western states (70.0%). These data suggest a geographical pattern in the distribution of AHVd, with higher detection rates in the southern and eastern states. The analysed subset of the screening samples contained in total 51 different apple varieties. All varieties showed at least one AHVd positive sample, except for six cultivars (Supplementary Table 2). For 28 of these varieties (55%) an infection rate of 100% was observed, i.e., all samples from a specific cultivar showed to be positive for AHVd.

**Table 2:** Summary of AHVd detection results in various German states based on RT real-time PCR and Sanger sequencing.

State	Total samples	AHVd negative	AHVd positive	Percentage of AHVd infections	AHVd positive reads	AHVd positive reads (%)
Baden-Wuerttemberg	35	7	28	80.0%	126	45%
Bavaria	19	3	16	84.2%	51	34%
Brandenburg	12	0	12	100.0%	34	35%
Hamburg	6	4	2	33.3%	2	4%
Hesse	14	0	14	100.0%	69	62%
Mecklenburg-Vorpommern	12	5	7	58.3%	30	31%
Lower Saxony	8	4	4	50.0%	16	25%
North Rhine-Westphalia	16	12	4	25.0%	12	9%
Rhineland-Palatinate	12	1	11	91.7%	32	33%
Saarland	8	2	6	75.0%	20	31%
Saxony	6	1	5	83.3%	27	56%
Saxony-Anhalt	16	0	16	100.0%	74	58%
Schleswig-Holstein	12	1	11	91.7%	46	48%
Thuringia	16	3	13	81.3%	62	48%
Total	192	43	149	77.6%	601	39%

Samples with at least one positive Sanger sequence were considered positive for AHVd (column “AHVd positive”). Further, number of positive reads and the percentage of AHVd positive reads relative to the possible maximum of eight reads per sample are presented for each state.

As a final test, tree age was evaluated as a potential factor influencing AHVd infection status. While age data were available for most samples, no significant correlation was found between tree age and infection (Supplementary Figure 1).

### Phylogenetic analysis

**Figure 1:**
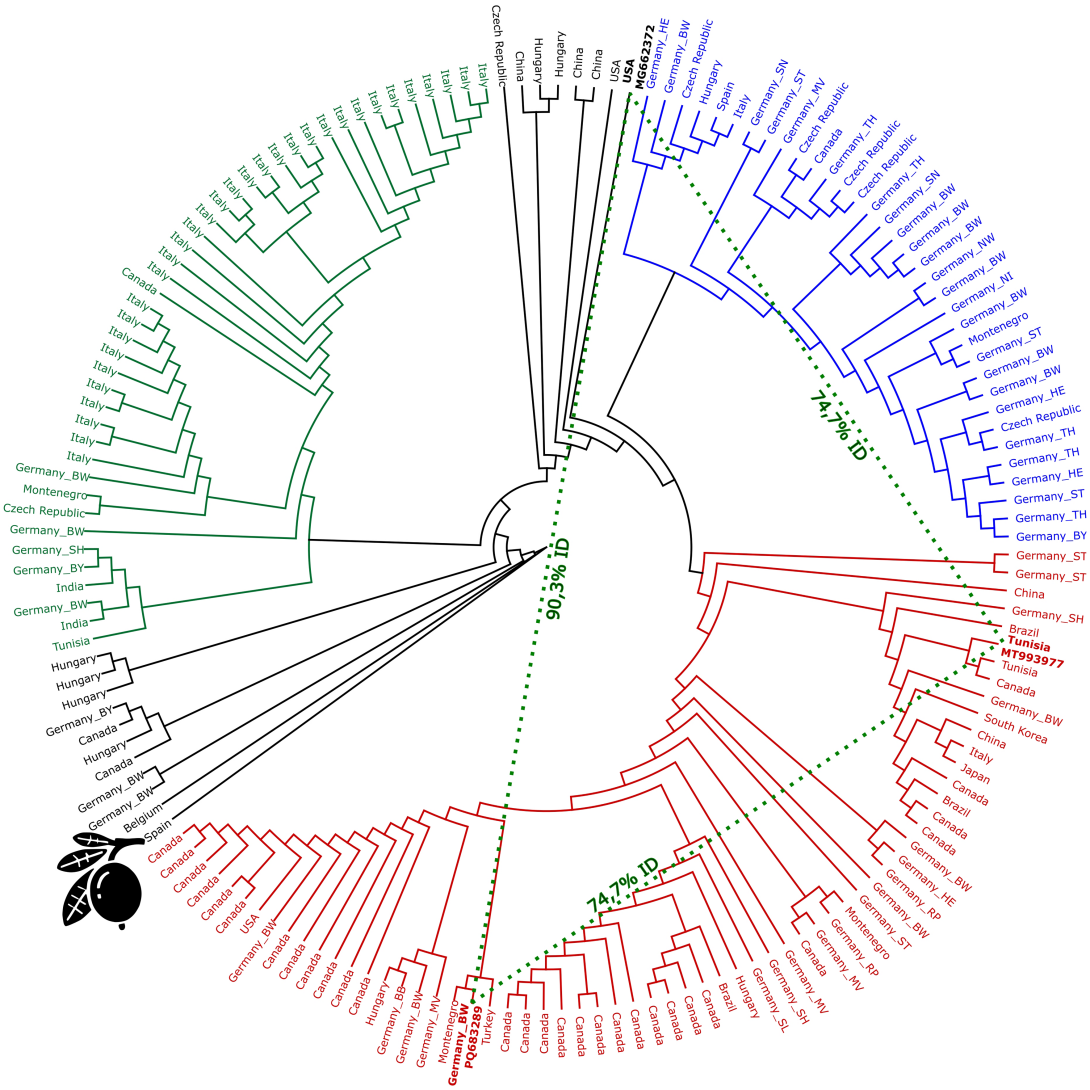
Phylogenetic tree of apple hammerhead viroid (AHVd) sequences. The tree was constructed using RAxML (v8.2.11) with the GTR GAMMA nucleotide model and the rapid hill-climbing algorithm, performing 100 bootstrap replicates. The clusters are color-coded based on predominant geographical associations: green represents the Italian cluster, blue the German cluster, and the Canadian cluster is red. The loquat icon indicates the according Spanish accession (OK272503). Dashed lines highlight the distant relationship between the Tunisian accession MT993977, the German accession PQ683289, and the USA accession MG662372, since those accessions show the highest distance within the apple-derived sequences.

The AHVd sequences analysed in this study, comprising isolates from *Malus domestica* (apple) and a single sequence from *Eriobotrya japonica* (loquat), formed three major clades, with individual clusters dominated by (a) Italian isolates (green), (b) Canadian isolates (red), and (c) German isolates (blue) (Figure 1). Additionally, some sequences from various origins, including China, Japan, Tunisia, Brazil, and South Korea, were interspersed across these clusters, underscoring the global distribution of AHVd. The Italian cluster consists primarily of Italian accessions, with some German samples included. This group shows relatively low substitution rates (under 0.02 substitutions per site, Supplementary Figure 2), suggesting recent common ancestry or ongoing gene flow within these regions. The North American cluster includes mostly sequences from Canada and the USA, along with a few from Brazil and Germany. This clade shows higher substitution rates (0.04–0.10 substitutions per site), indicating greater genetic divergence. The German cluster is dominated by German accessions but also includes some sequences from other European sources. This cluster exhibits relatively low substitution rates (below 0.03 substitutions per site), indicating closer genetic relationships among German sequences.

The loquat-derived sequence (OK272503) is notably shorter, with a length of 376 nt compared to the typical 433 nt average of other AHVd sequences. Among the apple-derived sequences, the maximum genetic distance observed was between MT993977 (from the red cluster, Tunisia) and both MG662372 (USA) and the newly identified German sequence PQ683289 (Baden-Wuerttemberg), with only 74.7% identity. It is important to note that PQ683289 and MG662372 share only a similarity of 90.3%.

### Genetic variation and small RNAs

**Figure 2:**
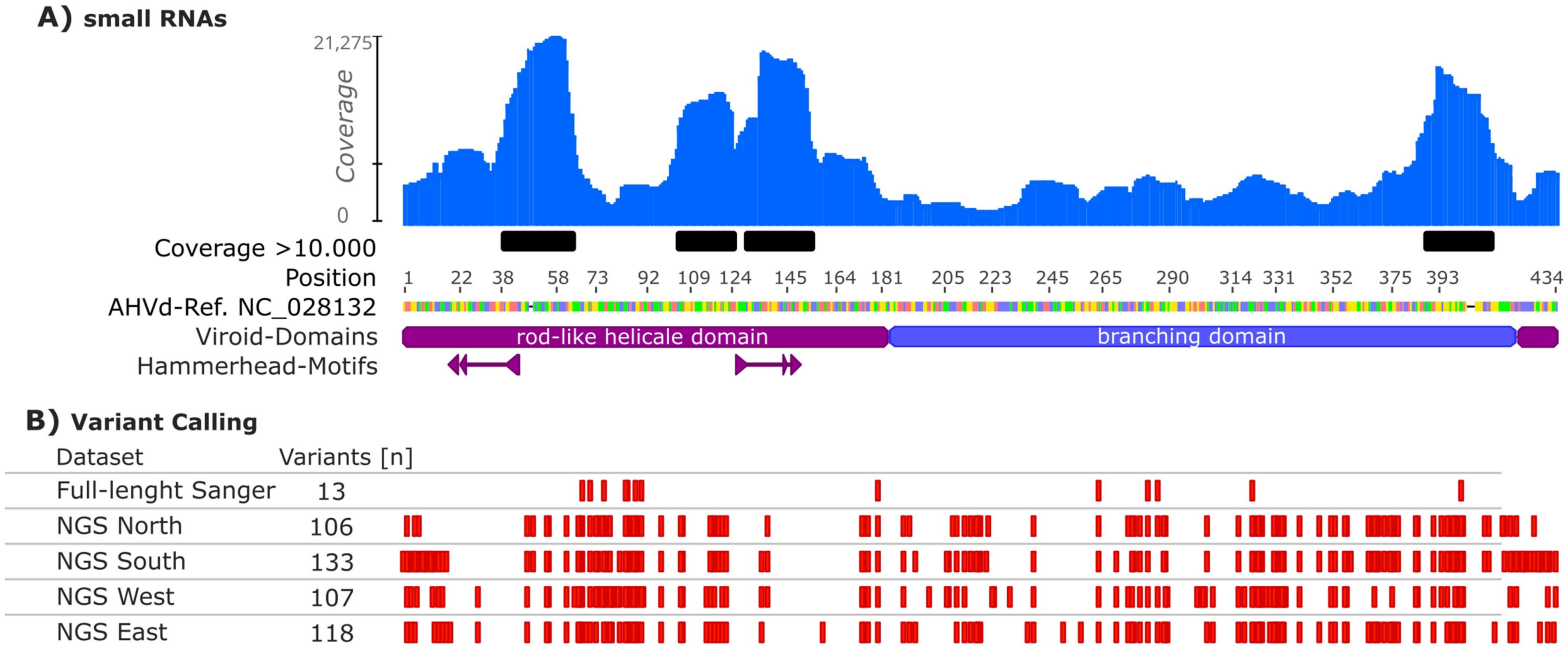
Coverage and variant analysis of the apple hammerhead viroid (AHVd) reference genome (NC_028132). **(A)** Coverage of small RNAs of a single tree from the apple cultivar “Nicoter” mapped to the AHVd genome, with regions exceeding 10,000× coverage highlighted in black. **(B)** Variant calling analysis including inter-sample variability based on Sanger sequencing (full-length Sanger), and inter-sample variability from NGS pools (ribo-depleted total RNA sequencing) grouped by Germany’s federal states (North, South, West, East). The rod-like helical domain of AHVd and hammerhead structures are indicated in purple, while the branching domain is labelled in blue. Variants are represented as red marks along the genome, with the total number of variants indicated for each dataset.

The AHVd genome’s variability was assessed across different datasets to capture inter-sample diversity (Figure 2B). Small RNA sequences (18–30 nt) from a single apple tree of the cultivar “Nicoter” were mapped to the AHVd reference genome (NC_028132, Figure 2A). Of the 29 million reads, 128,388 (approximately 0.44%) mapped to the AHVd reference sequence (Supplementary Table 3). These small RNAs accumulated at specific regions, primarily within the rod-like helical domain of the genome. Coverage was non-uniform, with four regions exceeding 10,000× coverage (Figure 2A). Inter-sample variability was evaluated through Sanger sequencing of full-length AHVd genomes from multiple trees, identifying 13 variable positions distributed across the genome (Figure 2B).

A comprehensive assessment of AHVd diversity was performed using ribo-depleted total RNA sequencing from pooled samples grouped by Germany’s federal states (North, South, West, East). Each regional pool yielded between 20 and 26 million paired-end reads (2 × 150 bp), with on average 0.12% of reads mapping to AHVd reference sequences, providing sufficient depth for reliable variant detection (Supplementary Table 3). These datasets identified 106 to 133 variable positions per pool, with substantial overlap across all pools. Of these, 44 positions were unique to individual pools, while 125 were shared among at least two pools, highlighting a significant degree of overlapping diversity. The hammerhead-motif regions in all datasets were conserved, except for position 19 of the consensus where a U to A transition was observed in 13% of the NGS East samples. Notably, a weak correlation of *R*^2^ = 41% was found between the number of samples and the number of variable positions.

## Discussion

AHVd was first detected in Germany in 2020 in two apple orchards in Baden-Wuerttemberg (Lake Constance district and Ortenau district; Zikeli et al., 2021). Noteworthy, along with AHVd, apple scar skin viroid (*Apscaviroid cicatricimali*, member of the *Pospiviroidae* family, genus *Apscaviroid*) was also first reported in apple in Germany (Zikeli et al., 2021).

The current study investigates the genetic variation and geographical distribution of AHVd in apple orchards across Germany. By combining RT real-time PCR and Sanger sequencing, we aimed to assess the prevalence of AHVd and explore its evolutionary lineage through phylogenetic analysis. Additionally, the study delves into inter-sample variability of AHVd sequences and identifies potential hotspots for viroid-derived small RNAs.

The high overall detection rate of AHVd (77.6%) across various German federal states indicates a widespread presence of the viroid in apple orchards. This is reflected in the high number of varieties (55%) of which all samples were tested AHVd positive. While the data suggests a geographical pattern with higher detection rates in the southern (82%) and eastern states (90%) compared to the northern (72%) and western states (70%), caution must be exercised in interpreting these results due to the relatively small sample size from each state, ranging from 17 to 53 samples, respectively. The variation in detection rates among different states could be attributed to regional differences in cultivation practices, environmental conditions, or the introduction of infected plant material, as AHVd is a graft-transmissible agent, effectively transmitted by vegetative propagation techniques such as grafting and budding.

A closer look at the breeding origin and parentage as well as distribution channels of planting material may provide insight into possible relationships. Among the cultivars with six or more analyzed samples, infection rates exceeding 70% were observed in the breeding varieties “Braeburn,” “Elstar,” “Jonagold,” “Pinova,” “Topaz,” “Rubinette,” “Wellant,” “Golden Delicious,” “breeding clone DE,” and “Shampion” (Supplementary Table 2). A common trait among these cultivars is their genetic lineage. Most are direct or suspected descendants of “Golden Delicious” and “Cox Orange,” two ancestor varieties known for their high susceptibility to fungal, bacterial, and viral diseases (Bannier, 2011). In particular, “Braeburn” and “breeding clone DE” are thought to share this ancestry, though their exact parentage remains uncertain.

Highest infection rates (100%, at least six samples analysed) were identified for “Golden Delicious,” “breeding clone DE” and “Shampion” (Supplementary Table 2). For the “breeding clone DE” a parentage of “Honeycrisp” is assumed. Interestingly, a “Honeycrisp” variety was first reported to be infected with AHVd in the USA (Szostek et al., 2018). Planting material of the “breeding clone DE” trace back to a single source, which is reflected in phylogenetic analysis of obtained AHVd sequences. AHVd isolates from “breeding clone DE” samples share a high sequence similarity, thus clustering in one phylogenetic group and emphasizing one genetic source (Supplementary Figure 2). As AHVd genomes of “Braeburn” samples were distributed across multiple clusters, this suggests distinct evolutionary origins (Supplementary Figure 2). Variant calling confirmed low variability in “breeding clone DE” and higher variability in “Braeburn,” “Shampion,” and “Elstar” (Supplementary Figure 3).

In contrast to the above-mentioned varieties the cultivar “Gala” as a New Zealand breed with a comparatively low infection rate of 56%, does not fit into this pattern despite its parentage including “Golden Delicious” and “Cox Orange” [“Kidds Orange” (“Red Delicious” × “Cox Orange”) × “Golden Delicious”; Bannier, 2011]. Despite its lower infection rate, “Gala” clustered with “Jonagold,” “Pinova,” and “Rubinette” and showed similarly high AHVd sequence variability, suggesting elevated mutation rates (Supplementary Table 2 and Supplementary Figure 2). It is noteworthy, that for the “old variety” “Boskoop” a lower infection rate of 55% and a relatively low sequence variability was recorded, as it was for the variety “Topaz,” possibly due to a missing long breeding history or mainly small-scale non-intensive orchards. An interruption of the spread of viroids can be achieved by testing planting material and selling healthy propagation material.

Understanding additional transmission mechanisms of AHVd in apple is essential to explain its high prevalence across diverse varieties and widespread distribution in Germany, spanning various locations, orchard types, fruit-growing farms, and variety collections, and to develop effective control measures. One possibility is mechanical transmission by contaminated tools, in analogy to other members of the genus *Pelamoviroid* (e.g., PLMVd) (Hadidi et al., 2022), although this mechanism is considered inefficient in woody hosts (Bragard et al., 2019).

Ongoing experiments at JKI institute on mechanical transmission of AHVd via pruning tools in apple trees and *in vitro* apple cultures have so far provided no evidence of transmissibility in apple (unpublished). Additional natural transmission mechanisms (insect vector, pollen, seed) cannot be excluded, according to the pest categorization report, due to analogies with other members of the same genus and family (Bragard et al., 2019). Current results of seed experiments at JKI institute provide no indication for seed transmission of AHVd in apple - in total 162 apple seedlings, 76 of cultivar “breeding clone DE” and 86 of cultivar “Mairac,” germinated from 199 seeds of AHVd infected fruits (pericarp tested positive with AHVd-13F_PG/AHVd-12R_PG, Messmer et al., 2017) and were tested negative for AHVd. The screening data obtained in this study could promote a consideration of AHVd in phytosanitary regulations and certification of planting material as the most effective control measures. However, reliable diagnostics, which are essential for implementing any regulations, remain a challenge.

This study addresses at least two questions raised by the EFSA in its 2019 pest categorization, the geographical distribution and prevalence of AHVd in the EU and the current availability of a comprehensive detection assay capable of detecting a wide range of AHVd isolates (Bragard et al., 2019). The technical learning from this analysis is that RT real-time PCR was able to enrich AHVd template for sequencing while not producing reliable amplification curves. We hypothesized that the high genetic variability between the different samples, i.e., inter-sample variability, leads to different curve shapes per sample and even between runs, making the RT real-time PCR results inconsistent. However, our approach of combining RT real-time PCR and Sanger sequencing is costly and impractical for standard high-throughput screening. Immunoassays are not suitable for viroid RNAs (Zhang and Li, 2024). Therefore, further research is needed to develop a robust yet inexpensive method for AHVd detection. Recent developments in CRISPR Cas13a systems for RNA detection, such as the SHERLOCK platform, have shown promise for viroid detection (Zhai et al., 2024). The SHERLOCK platform may be particularly effective because it requires only a single short target sequence, which could be one of the few highly conserved regions of the AHVd genome.

The phylogenetic tree analysis of AHVd sequences branches into clusters associated with distinct geographical origins, which may reflect evolutionary lineages among the sequences (Figure 1). The Italian cluster (green) and the North American cluster (red) suggest isolated evolutionary paths for AHVd in these regions, possibly due to limited exchange of plant material or localized spread of the viroid. This is consistent with previous findings by Chiumenti et al. (2019), who reported that Italian accessions cluster together, in contrast to North American accessions and others. Additionally, Messmer et al. (2017) reported a clear phylogenetic distinction between two origins for their Canadian apple cultivars tested, and indeed the North American cluster in our analysis has two major branches, reflecting this finding (Figure 1). Some accessions could not be assigned to any cluster, including sequences from various countries, such as Hungary, Germany, China, and Belgium, suggesting a more intricate evolutionary history. Sanderson and James (2019) highlighted the genetic diversity of AHVd and its widespread distribution, supporting the idea of complex transmission pathways across different geographical areas. Generally, German accessions are dispersed throughout the tree, appearing in each cluster, indicating no clear pattern of geographical or evolutionary isolation. Analyzing the German accessions solely did not reveal clear geographical clustering, considering the states or even north-to-south or west-to-east comparisons. This widespread distribution within the tree might suggest frequent and diverse introduction events or extensive local spread within Germany. It may also result from a more diverse environment or cultivars, leading to a higher degree of viroid adaptation.

However, without further viroid adaptation experiments, these questions will be difficult to resolve since drawing conclusions from viroid sequences to their geographical origin or evolution might be much more challenging compared to viruses and other organisms with larger genomes. For example, a study on the effect of high temperatures (1 week starting at 34°C and increasing it to 40°C daily) on the hop latent viroid showed that even a relatively short period of warm temperatures can boost viroid quasispecies variability, even for a highly conserved viroid such as the potato spindle tuber viroid (Matoušek et al., 2004). Considering that the *Avsunviroidae*, such as AHVd, are among the most genetically variable entities on earth (López-Carrasco et al., 2017), it is almost surprising that phylogenetic information can be recovered. However, some functionally important regions remain conserved, even among hammerhead viroids.

The Sanger sequencing reads were used not only for screening but also re-analyzed to assess the overall inter-sample polymorphisms, resulting in a low variability of 3.0% of the viroid’s genome. This appears low compared to the 12–18% AHVd variability reported by Chiumenti et al. (2019) for international variants, and the 50% variability observed in PLMVd by Glouzon et al. (2014). However, it must be considered that these studies used NGS data, while Sanger sequencing typically covers only the dominant sequence variations of a quasispecies (Knyazev et al., 2021). All variants identified through Sanger sequencing in this study were also represented in the NGS data (Figure 2B), suggesting that Sanger sequencing captures the most widespread or conserved variants within the German AHVd population. In addition, the low variability assessed via Sanger sequencing of RT real-time PCR products demonstrates a higher selectivity of RT-PCR compared to unbiased approach of NGS analysis. Messmer et al. (2017) stated that NGS is less reliable than RT-PCR, but their study used polyadenylated RNA enrichment for NGS-library preparation. Since viroids lack mRNA they do not have polyadenylated-tails and do not get enriched (Pecman et al., 2017). In our study, we followed the approach of Pecman et al. (2017) and applied ribosomal RNA depletion in combination with RNA sequencing for both small RNA and total RNA analyses.

The NGS data for inter-sample variability covered the diversity across NGS pools and highlighted regional viroid population variability. Total RNA-based ribo-depleted sequencing revealed a substantial number of variable positions (106–133 per pool), indicating diverse AHVd populations in Germany’s apple-growing regions. These findings align with reports of high AHVd diversity in geographically distant regions, such as Italy, the USA, and India (Szostek et al., 2018; Nabi and Baranwal, 2020). In our study, it was observed that sample size influenced variability, with the South pool exhibiting the highest variability and the North pool the least, reflecting the differing contributions of individual samples to the pools.

Interestingly, 125 variable positions were shared across at least two pools, while 44 positions were unique to individual pools. These unique positions may represent localized genetic variants shaped by regional viroid evolution or specific cultivar compositions, as previously noted for AHVd’s distribution in global studies (Lim et al., 2019). Overlapping positions likely reflect common evolutionary pressures, consistent with AHVd’s association with frequent exchange of apple material across regions. Summing all German AHVd variable positions identified in this study yields 169, equalling 39% sequence variability within the German AHVd population. Despite this high overall genetic variability, the conserved hammerhead structures essential for viroid replication exhibited almost no polymorphisms, underscoring their critical functional role. Only one variable position (position 19) interferes with the hammerhead motif in the NGS East pool (Figure 2). Similarly, López-Carrasco et al. (2017) reported significant genetic diversity in Eggplant latent viroid (ELVd), while key structural motifs like hammerhead ribozymes remained highly conserved due to evolutionary pressures to maintain functional integrity. These findings highlight the balance between adaptability and structural conservation in viroid genomes.

For plant pathologists, however, this extreme genomic flexibility complicates detection in routine diagnostics, emphasizing the need for adaptable and comprehensive detection strategies.

The small RNA data analysis identified potential hotspots for vd-sRNAs (Figure 2). Coverage analysis highlighted regions with coverage above 10,000, indicating areas of high small RNA activity. These hotspots were located both within the rod-like part of the AHVd genome and outside this region, suggesting that vd-sRNAs may contribute to both structural and regulatory functions of the viroid. While vd-sRNAs have not been discussed for AHVd to our knowledge, Di Serio et al. (2009) conducted deep sequencing of small RNAs derived from two symptomatic variants of peach latent mosaic viroid (PLMVd). In their study, multiple vd-sRNAs were identified, with a prevalence of 21-nt (+) and (−) RNAs displaying a biased distribution of their 5′ nucleotide and adopting a hotspot profile along the genomic (+) and (−) RNAs.

Our analysis indicates that the distribution of hotspots in the AHVd genome mirrors the findings of Di Serio et al., where the hotspot patterns of vd-sRNAs did not map solely to the expected hammerhead arm regions but rather to areas forming multiple shorter hairpins. This pattern suggests that the viroid genomic RNAs alone do not account for the observed vd-sRNA profile, and that secondary RNA structures and processing by host dicer-like proteins are significant factors. Consequently, the presence of vd-sRNA might be part of the pathogenesis of *Avsunviroidae*, as it is for *Pospiviroidae*, where vd-sRNAs are associated with silencing host transcripts and leading to RNA-guided methylation, which ultimately results in viroid symptoms (Hammann and Steger, 2012). Further research into the functional roles of these small RNAs will unravel the specific biology of these highly variable smallest pathogens of the genus *Avsunviroidae*.

## Conclusion

This study highlights the substantial genetic variability between AHVd samples, with overall sample variability reaching 39%, akin to findings in other *Avsunviroidae* members such as PLMVd. Despite the variability, crucial motifs for the hammerhead ribozyme remain conserved, suggesting evolutionary pressures to maintain functional integrity. The widespread presence of AHVd across German apple orchards, with higher detection rates in southern and eastern states, indicates a broad distribution influenced by regional cultivation practices and environmental conditions. Phylogenetic analysis reveals distinct clusters based on geographical origin, hinting at isolated evolutionary paths and complex transmission histories. Additionally, the identification of vd-sRNAs hotspots within the AHVd genome suggests roles in both structural and regulatory functions, potentially contributing to viroid pathogenesis. However, the high genetic diversity poses challenges for diagnostic screening, necessitating the development of advanced tools like CRISPR-Cas13a systems. For apple producers, the clear risk remains unresolved due to the difficulty in correlating specific symptoms with AHVd variants, emphasizing the need for comprehensive monitoring and management strategies including the possible inclusion in phytosanitary regulations and certification schemes, and further research to elucidate the molecular mechanisms of viroid infections.

## Data Availability

The small RNA sequencing data were submitted to the European Nucleotide Archive (ENA) under study accession PRJEB88384 (run accession ERR14838686).
