# Supplementary material for: Prevalence, genetic diversity, and molecular detection of the apple hammerhead viroid in Germany
Source: Front Microbiol. 2025 Jun 3;16:1592572. doi: 10.3389/fmicb.2025.1592572 (PMC12170603; doi:10.3389/fmicb.2025.1592572)
Supplement: Supplementary file 1 [file Table_1.pdf]

Prevalence, genetic diversity, and molecular detection of the *apple hammerhead viroid* in Germany

Kerstin Zikeli[1], Constanze Berwarth[1], Ute Born[2], Thomas Leible[1], Wilhelm Jelkmann[1], Michael Helmut Hagemann [2]

[1] Julius Kühn-Institute, Federal Research Centre for Cultivated Plants, Institute for Plant Protection in Fruit Crops and Viticulture, Schwabenheimer Str. 101, 69221 Dossenheim, Germany

[2] University of Hohenheim, Production Systems of Horticultural Crops, Emil-Wolff-Str. 25, 70599 Stuttgart, Germany

Supplemental Tables

Supplemental Table 1. List of primers and probes used in this study to achieve full length sequences.

Primer	Sequence	Author
AHVd_IT_R	TGTGATCCCGTTGGATCTCAC	This publication
AHVd_MHxF	ACCCCTCCGGTCTTATCCAA	This publication
AHVd_MHxR	CGTCCTTGGAACGGACTCAT	This publication
AHVd_RF-1379	CUUAUCCAACCUCUGUUUCGGCAGAGGAUAC	Serra et al., 2018
AHVd_RF-1380	GGAACUAGGGAACCCCUCCGGU	Serra et al., 2018
AHVd_RF-1385	CUCUCUCGCCCUAUACAUCCUCU	Serra et al., 2018
AHVd_RF-1386	CAGGCAUAGAGAUGACCAUUUGC	Serra et al., 2018
AHVd-1	TTAGCCTTCCTGATGAGTCC	Lim et al., 2019
AHVd-12R_PG	CTAATAGCCTCCGACCGTCAT	Messmer et al., 2017
AHVd-13F_PG	CCTTCCTGATGAGTCCGTTCCA	Messmer et al., 2017
AHVd-2	TGTGTCTACTTAAAGACTCAC	Lim et al., 2019
AHVd-3R	GTCCTTTTAGGACGAAACTT	This publication
AHVd-4F	TCATCAGGTAGCCTAATAGACTA	This publication
AHVd-5R	GAGAGAGAGCGACTTCTCTC	This publication
AHVd-6F	TATACATCCTCTCAGG	This publication
AHVd-88F_PG	TAGTTACTTCCGGTAACTTGGA	Messmer et al., 2017
AHVd-CH_F	CCAAGGACGAAACCCG	This publication
AHVd-CH_R	CCAAGGACGAAACCG	This publication
AHVd-ZK-P_HEX	(HEX)TCATCAGGTAGCCTAATAGACTAC(BHQ1)	This publication
