# Supplementary material for: Prevalence, genetic diversity, and molecular detection of the apple hammerhead viroid in Germany
Source: Front Microbiol. 2025 Jun 3;16:1592572. doi: 10.3389/fmicb.2025.1592572 (PMC12170603; doi:10.3389/fmicb.2025.1592572)
Supplement: Supplementary file 2 [file Table_2.pdf]

Prevalence, genetic diversity, and molecular detection of the *apple hammerhead viroid* in Germany

Kerstin Zikeli[1], Constanze Berwarth[1], Ute Born[2], Thomas Leible[1], Wilhelm Jelkmann[1], Michael Helmut Hagemann [2]

[1] Julius Kühn-Institute, Federal Research Centre for Cultivated Plants, Institute for Plant Protection in Fruit Crops and Viticulture, Schwabenheimer Str. 101, 69221 Dossenheim, Germany

[2] University of Hohenheim, Production Systems of Horticultural Crops, Emil-Wolff-Str. 25, 70599 Stuttgart, Germany

Supplemental Table

Supplemental Table 2. Detection of apple hammerhead viroid (AHVd) across different apple cultivars. The table summarizes the number of samples per cultivar that tested negative (No Detection) or positive (AHVd Detected) for AHVd, along with the total number of analysed samples (Sum) and the percentage of AHVd-positive samples (Percent).

Apple Cultivar	No Detection	AHVd Detected	Sum	Percent
Braeburn	3	16	19	84%
Gala	7	9	16	56%
Elstar	2	12	14	86%
Jonagold	3	11	14	79%
Boskoop	5	6	11	55%
Pinova	2	7	9	78%
Topaz	2	6	8	75%
Rubinette	2	5	7	71%
Wellant	2	5	7	71%
Golden Delicious	0	6	6	100%
breeding clone DE	0	6	6	100%
Shampion	0	6	6	100%
Red Jonaprince	1	4	5	80%
Fuji	0	4	4	100%
Kanzi	2	2	4	50%
Adersleber Kalvill	0	3	3	100%
Delbarestivale	0	3	3	100%
Schöner aus Nordhausen	0	3	3	100%
Barbarossa	0	2	2	100%
Berlepsch	0	2	2	100%
Bittenfelder	1	1	2	50%
Doppelter Prinzenapfel	0	2	2	100%
Evelina	0	2	2	100%
Gravensteiner	1	1	2	50%
Holsteiner Cox	1	1	2	50%
Idared	0	2	2	100%

Jonagored	1	1	2	50%
Mariella	0	2	2	100%
Nicoter	0	2	2	100%
Santana	0	2	2	100%
Seestermüher Zitronenapfel	1	1	2	50%
Wurtwinning	1	1	2	50%
Delbare	0	1	1	100%
Filippa	0	1	1	100%
Florina	1	0	1	0%
Galmac	0	1	1	100%
Haux	0	1	1	100%
Hilde	0	1	1	100%
Lord Lambourne	0	1	1	100%
Mairac	0	1	1	100%
Morgana	1	0	1	0%
Natyra	1	0	1	0%
Pirol	0	1	1	100%
Red Elstar	0	1	1	100%
Red Flame	0	1	1	100%
Remo	0	1	1	100%
Rene	1	0	1	0%
Rewena	1	0	1	0%
Rubinstar	0	1	1	100%
Ruby Frost	0	1	1	100%
Snap Dragon	1	0	1	0%
