# Supplementary material for: Prevalence, genetic diversity, and molecular detection of the apple hammerhead viroid in Germany
Source: Front Microbiol. 2025 Jun 3;16:1592572. doi: 10.3389/fmicb.2025.1592572 (PMC12170603; doi:10.3389/fmicb.2025.1592572)
Supplement: Supplementary file 3 [file Table_3.pdf]

Prevalence, genetic diversity, and molecular detection of the *apple hammerhead viroid* in Germany

Kerstin Zikeli[1], Constanze Berwarth[1], Ute Born[2], Thomas Leible[1], Wilhelm Jelkmann[1], Michael Helmut Hagemann [2]

[1] Julius Kühn-Institute, Federal Research Centre for Cultivated Plants, Institute for Plant Protection in Fruit Crops and Viticulture, Schwabenheimer Str. 101, 69221 Dossenheim, Germany

[2] University of Hohenheim, Production Systems of Horticultural Crops, Emil-Wolff-Str. 25, 70599 Stuttgart, Germany

Supplemental Table

Supplemental Table 3. Overview of sequencing datasets and read counts matching the apple hammerhead viroid (AHVd). The small RNA library from 'Kanzi' was used to identify viroid-derived small RNAs. Four additional ribo-depleted RNA-seq libraries were generated from pooled samples of apple trees representing different German regions. Matching reads refer to the number of reads aligning to AHVd reference sequences as determined by custom bioinformatic pipelines.

Sample ID	Library type	Read length	Raw reads	AHVd-matching reads
P01-H02-Kanzi-1	small RNA (size-selected)	1×75 bp	29,023,526	128,388
AHVd-North	ribo-depleted RNA-seq	2×150 bp	24,458,766	23,334
AHVd-East	ribo-depleted RNA-seq	2×150 bp	20,554,908	18,083
AHVd-South	ribo-depleted RNA-seq	2×150 bp	21,055,224	45,385
AHVd-West	ribo-depleted RNA-seq	2×150 bp	25,655,512	22,597
