# Supplementary material for: Prevalence, genetic diversity, and molecular detection of the apple hammerhead viroid in Germany
Source: Front Microbiol. 2025 Jun 3;16:1592572. doi: 10.3389/fmicb.2025.1592572 (PMC12170603; doi:10.3389/fmicb.2025.1592572)
Supplement: Supplementary file 5 [file Image_1.pdf]

Prevalence, genetic diversity, and molecular detection of the *apple hammerhead viroid* in Germany

Kerstin Zikeli[1], Constanze Berwarth[1], Ute Born[2], Thomas Leible[1], Wilhelm Jelkmann[1], Michael Helmut Hagemann [2]

[1] Julius Kühn-Institute, Federal Research Centre for Cultivated Plants, Institute for Plant Protection in Fruit Crops and Viticulture, Schwabenheimer Str. 101, 69221 Dossenheim, Germany

[2] University of Hohenheim, Production Systems of Horticultural Crops, Emil-Wolff-Str. 25, 70599 Stuttgart, Germany

Supplemental Figure

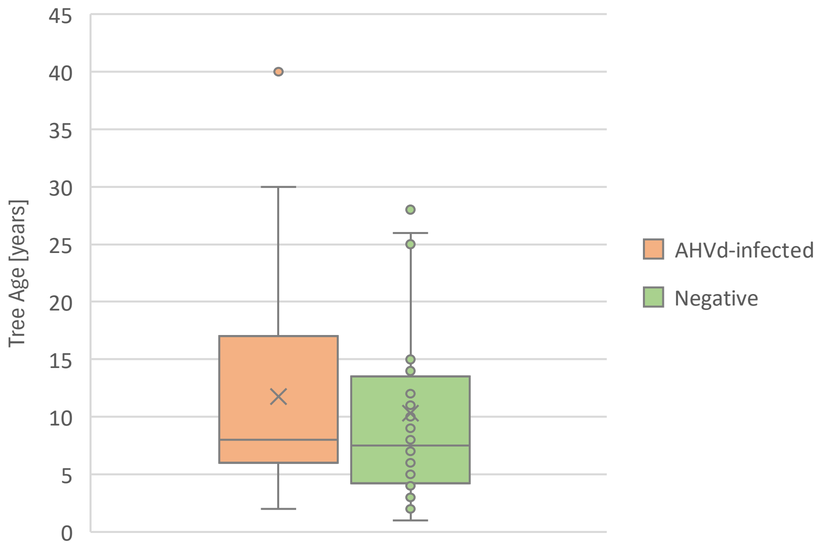

Supplemental Figure 1. Relationship between tree age and apple hammerhead viroid (AHVd) infection status in German apple cultivars. Tree age distribution is shown for AHVd-positive (n = 134) and AHVd-negative (n = 40) apple trees. Only trees with known age and AHVd status were included. Although older trees appear slightly more likely to test positive, the difference in mean age between infected and uninfected trees was not statistically significant (Welch's t-test, p = 0.3393). This suggests that AHVd infection is not strongly dependent on tree age.
