# Supplementary material for: Prevalence, genetic diversity, and molecular detection of the apple hammerhead viroid in Germany
Source: Front Microbiol. 2025 Jun 3;16:1592572. doi: 10.3389/fmicb.2025.1592572 (PMC12170603; doi:10.3389/fmicb.2025.1592572)
Supplement: Supplementary file 6 [file Image_2.pdf]

Prevalence, genetic diversity, and molecular detection of the *apple hammerhead viroid* in Germany

Kerstin Zikeli[1], Constanze Berwarth[1], Ute Born[2], Thomas Leible[1], Wilhelm Jelkmann[1], Michael Helmut Hagemann [2]

[1] Julius Kühn-Institute, Federal Research Centre for Cultivated Plants, Institute for Plant Protection in Fruit Crops and Viticulture, Schwabenheimer Str. 101, 69221 Dossenheim, Germany

[2] University of Hohenheim, Production Systems of Horticultural Crops, Emil-Wolff-Str. 25, 70599 Stuttgart, Germany

Supplemental Figure

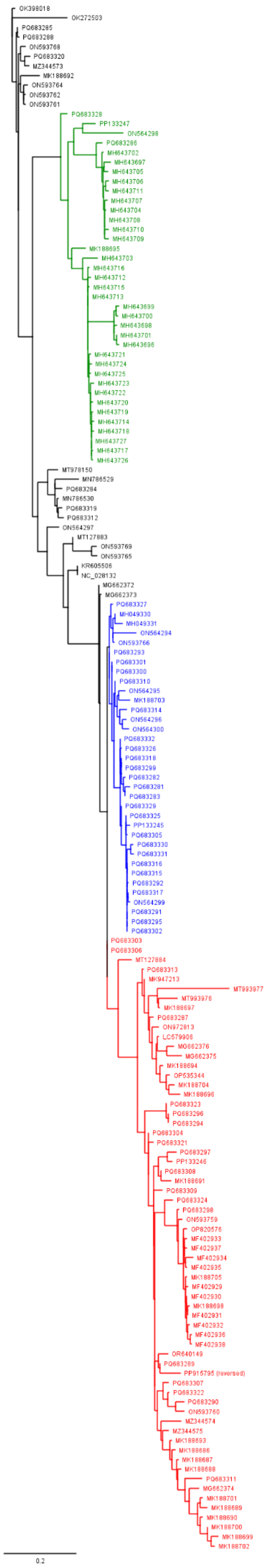

Supplemental Figure 2. Linear representation of the phylogenetic tree of apple hammerhead viroid (AHVd) sequences, constructed using RAxML (v8.2.11) with the GTR GAMMA nucleotide model and the rapid hill-climbing algorithm, with 100 bootstrap replicates. This tree is an alternative representation of Figure 1, allowing researchers to extract accession numbers and assess sequence substitutions per site. Clusters are color-coded by predominant geographical association: green for Italian, blue for German, and red for Canadian isolates. The Spanish loquat-derived accession (OK272503) is included as an outgroup. The scale bar represents nucleotide substitutions per site. Metadata and sequences are supplied by the file Supplementary_Table_Sample_Metadata.xlxs .
