# Supplementary material for: Prevalence, genetic diversity, and molecular detection of the apple hammerhead viroid in Germany
Source: Front Microbiol. 2025 Jun 3;16:1592572. doi: 10.3389/fmicb.2025.1592572 (PMC12170603; doi:10.3389/fmicb.2025.1592572)
Supplement: Supplementary file 7 [file Image_3.pdf]

Prevalence, genetic diversity, and molecular detection of the *apple hammerhead viroid* in Germany

Kerstin Zikeli[1], Constanze Berwarth[1], Ute Born[2], Thomas Leible[1], Wilhelm Jelkmann[1], Michael Helmut Hagemann [2]

[1] Julius Kühn-Institute, Federal Research Centre for Cultivated Plants, Institute for Plant Protection in Fruit Crops and Viticulture, Schwabenheimer Str. 101, 69221 Dossenheim, Germany

[2] University of Hohenheim, Production Systems of Horticultural Crops, Emil-Wolff-Str. 25, 70599 Stuttgart, Germany

Supplemental Figure

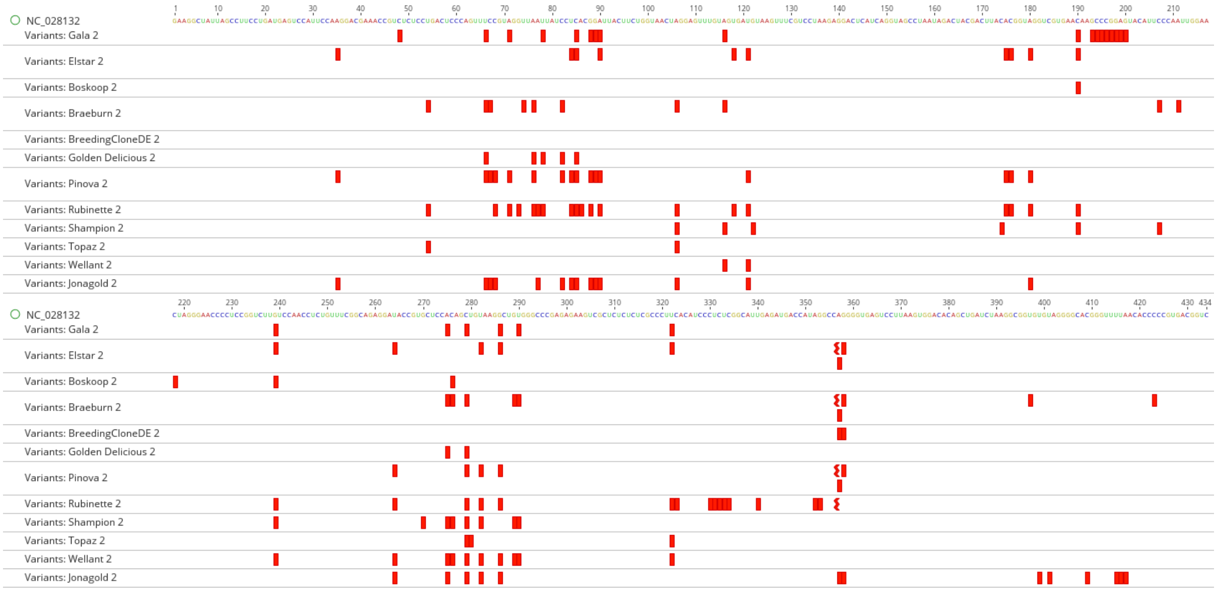

Supplemental Figure 3. Sequence variability of apple hammerhead viroid (AHVd) across different apple cultivars. Variant calling was performed per cultivar with at least six analyzed samples, using Geneious Prime. Mapping was conducted with the Geneious RNA mapper at highest sensitivity and default settings. Variants were identified using a minimum coverage of 4 and a minimum variant frequency of 0.3, while ignoring the reference sequence to capture within-sample variability. Red bars indicate nucleotide polymorphisms in comparison to the reference sequence NC_028132, highlighting distinct mutation patterns across cultivars. This representation provides insight into intra-host diversity and potential cultivar-specific selection pressures.
