# Supplementary material for: Prevalence, genetic diversity, and molecular detection of the apple hammerhead viroid in Germany
Source: Front Microbiol. 2025 Jun 3;16:1592572. doi: 10.3389/fmicb.2025.1592572 (PMC12170603; doi:10.3389/fmicb.2025.1592572)
Supplement: Supplementary file 8 [file Data_Sheet_1.pdf]

Prevalence, genetic diversity, and molecular detection of the *apple hammerhead viroid* in Germany

Kerstin Zikeli[1], Constanze Berwarth[1], Ute Born[2], Thomas Leible[1], Wilhelm Jelkmann[1], Michael Helmut Hagemann [2]

[1] Julius Kühn-Institute, Federal Research Centre for Cultivated Plants, Institute for Plant Protection in Fruit Crops and Viticulture, Schwabenheimer Str. 101, 69221 Dossenheim, Germany

[2] University of Hohenheim, Production Systems of Horticultural Crops, Emil-Wolff-Str. 25, 70599 Stuttgart, Germany

Supplemental File Overview

Item	Description	Format	Content
Supplementary Table 1	Primer and probe sequences used for RT real-time PCR and sequencing	Table (in this document)	19 primer and probe sequences with names, sequence, and author.
Supplementary Table 2	Detection of AHVd across apple cultivars	Table (in this document)	51 cultivars, with number of positive/negative samples per variety
Supplementary Table 3	Sequencing read statistics and AHVd-matching read counts	Table (in this document	Metadata of sequencing libraries with total reads and AHVd read counts
Supplementary Figure 1	Tree age vs. AHVd infection status in apple cultivars	Figure (in this document)	174 trees with known age and infection status, shown as boxplot
Supplementary Figure 2	Phylogenetic tree of AHVd full-length sequences	Figure (in this document)	RAxML tree with bootstrap values and sequence origin annotation
Supplementary Figure 3	Sequence variability of AHVd across cultivars	Figure (in this document)	Variant map per cultivar (subset); based on RT-PCR and NGS data
Supplementary Table Sample Metadata	Sample metadata including NCBI accession number, host plant and cultivar, country of origin and region, and sequence	Excel file	161 apple samples; includes sample ID, cultivar, region, infection status
